# Persistent Symptoms of Dengue: Estimates of the Incremental Disease and Economic Burden in Mexico

**DOI:** 10.4269/ajtmh.15-0896

**Published:** 2016-05-04

**Authors:** D. Carolina Tiga, Eduardo A. Undurraga, José Ramos-Castañeda, Ruth A. Martínez-Vega, Cynthia A. Tschampl, Donald S. Shepard

**Affiliations:** Instituto Nacional de Salud Pública, Centro de Investigaciones sobre Enfermedades Infecciosas, Cuernavaca, Mexico; Schneider Institutes for Health Policy, Heller School for Social Policy and Management, Brandeis University, Waltham, Massachusetts; Center for Tropical Diseases, University of Texas Medical Branch at Galveston, Galveston, Texas; Escuela de Microbiología, Universidad Industrial de Santander, Bucaramanga, Colombia

## Abstract

Dengue is mostly considered an acute illness with three phases: febrile, critical with possible hemorrhagic manifestations, and recovery. But some patients present persistent symptoms, including fatigue and depression, as acknowledged by the World Health Organization. If persistent symptoms affect a non-negligible share of patients, the burden of dengue will be underestimated. On the basis of a systematic literature review and econometric modeling, we found a significant relationship between the share of patients reporting persisting symptoms and time. We updated estimates of the economic burden of dengue in Mexico, addressing uncertainty in productivity loss and incremental expenses using Monte Carlo simulations. Persistent symptoms represent annually about US$22.6 (95% certainty level [CL]: US$13–US$29) million in incremental costs and 28.2 (95% CL: 21.6–36.2) additional disability-adjusted life years per million population, or 13% and 43% increases over previous estimates, respectively. Although our estimates have uncertainty from limited data, they show a substantial, unmeasured burden. Similar patterns likely extend to other dengue-endemic countries.

Dengue incidence and its geographical range have expanded substantially in the past decades; it has become a major public health challenge to most tropical and subtropical countries worldwide.^1^ Recent estimates suggest there are approximately 390 million dengue virus (DENV) infections annually, resulting in about 50–100 million symptomatic dengue episodes and 10,000 deaths.^1^–^3^ Several studies have examined the duration of symptomatic DENV infections.^4^,^5^ They typically last from 2 to 7 days, but may span a wide clinical spectrum.^2^ A symptomatic episode usually comprises a febrile phase (with fever of at least 38.5°C), a critical phase around defervescence (which may include hemorrhagic manifestations and/or dengue shock syndrome), and a recovery or convalescent phase.^2^ However, some dengue patients present persistent symptoms including fatigue, depression, and weight loss after the recovery phase, a possibility acknowledged by the World Health Organization (WHO) since 1997.^6^

A Malaysian study^4^ found that the adverse effects of symptomatic DENV infection on patients' quality of life (QoL) extend well beyond the febrile phase, although by day 14 of illness most patients in the sample had returned to a QoL of at least 90%. Fatigue, which results in decreased capacity to work, is common during the acute stage of dengue and may persist for several weeks after recovery.^2^,^7^ Nevertheless, most studies have focused on the acute manifestation of dengue illness. If persistent symptoms affect a non-negligible share of the population, previous studies have likely underestimated the burden of dengue.

As accurate estimates of disease and economic burden are critical to inform policy decisions and to assess technologies for dengue control and prevention, several authors have called for a broader evaluation of dengue burden.^8^,^9^ On the basis of a systematic literature review, we updated estimates of the economic burden of dengue in Mexico,^10^ addressing uncertainty in productivity loss due to persistent symptoms and in incremental expenses using Monte Carlo simulations.

We performed a systematic literature review on PubMed, MEDLINE, SciELO, and the WHO's Dengue Bulletin combining the keyword “dengue” with each of the following: fatigue, chronic, persist*, post-infect*, long-term, and clinical symptom*, for years 1995 through October 2015, in English, Spanish, French, and Portuguese (* indicates that additional letters were optional). We selected all articles related to persistent symptoms of dengue that had full text available, empirical data on potential work loss, a scientifically valid approach, and external validity. We excluded reviews, editorials, purely subjective papers, opinions, and duplicated studies (Supplemental Material, Supplemental Figure 1 and Supplemental Table 1).

Of 2,221 titles from the search, we identified 69 articles relevant for review and found 10 studies that satisfied our inclusion and exclusion criteria. The 10 studies were from Brazil, Cuba, Peru, and Singapore. Persistent symptoms were usually associated with female gender and older age and also generated belated medical expenses. Table 1 shows a summary of the most relevant studies providing empirical evidence of persistent symptoms after symptomatic DENV infection.^7^,^11^–^19^

We then extracted the proportions of patients that reported persistent symptoms that likely resulted in work loss, from 1 week to 2 years after hospital discharge (27 data points). Using these data, we examined the relationship between the share of patients (*S*) reporting difficulty to work, fatigue, or asthenia, and time elapsed following a symptomatic DENV infection in months (*T*), using Eq (1):

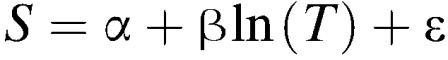
 where ln denotes the natural logarithm of the variable, α and β are coefficients, and ϵ is an error term. We found a significant relationship (*P* < 0.001, *R*^2^ = 0.43) between *S* and *T*, as shown in Figure 1

**Figure 1.**
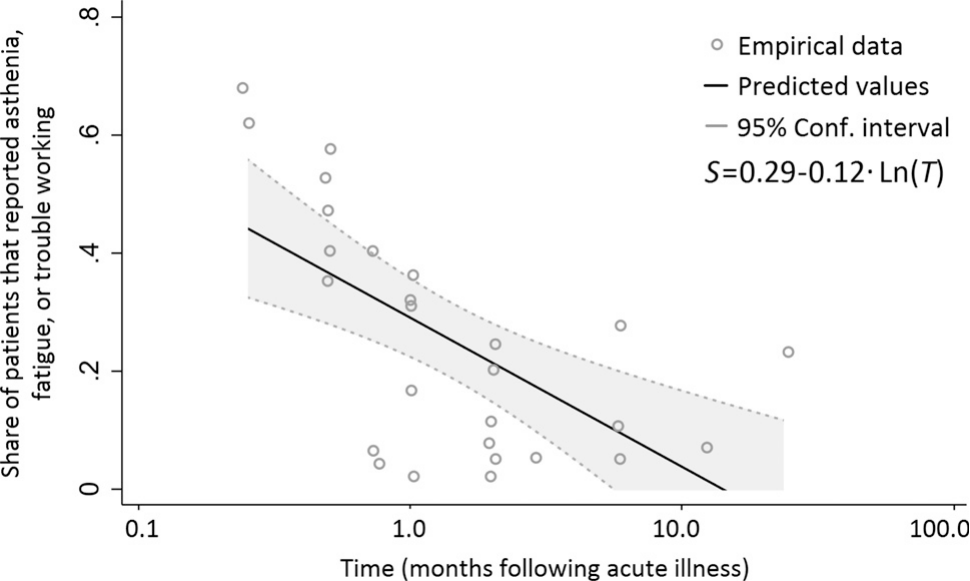
Association between the share of patients (*S*) reporting persistent symptoms that may result in work loss (fatigue, asthenia, or trouble working) and time (*T*) following an acute dengue virus (DENV) infection (in natural logarithm). *S* denotes the share of patients reporting difficulty to work, fatigue, or asthenia and *T* denotes the time elapsed following a symptomatic DENV infection in months.

.

We used the predicted values of persistent symptoms of dengue to extend recent estimates of the burden of dengue in Mexico, using the previously reported economic and disease burden parameters for acute dengue episodes.^10^ To address uncertainty in our estimates, we used a probabilistic sensitivity analysis. We allowed for variation in the main parameters (i.e., expansion factors, direct medical costs, direct nonmedical costs, health service utilization, patient impact, and household impact), and addressed uncertainty in the loss of productivity, additional expenses in medications, and diagnostic tests that may result from persistent symptoms, and disability weights. We computed 10,000 Monte Carlo simulations based on the simultaneous variation of all parameters in the model (Supplemental Table 2, Supplemental Material).

We added the following assumptions to those previously used.^10^ First, we assumed that 77% of patients spent some money in medication or diagnostic tests,^13^ and, based on the expenditures of symptomatic patients who did not seek health care in a health center or hospital in the Mexican study,^10^ we assumed that the monthly expenditures were US$10.52 (range: US$0.00–US$17.23; Beta-PERT distribution). Next, considering rates of household and work productivity loss and the probability of employment of people with chronic fatigue syndrome,^20^,^21^ we estimated that patients lose 45% of their working productivity (range: 15–65%; Beta-PERT distribution). Finally, we assumed that only adults were affected by persistent symptoms, because persistent symptoms were positively correlated with older age.^7^,^13^,^15^

Table 2 shows a summary of our cost-of-illness estimates, and the overall economic burden for Mexico. The results, in 2012 U.S. dollars with 95% certainty levels (CLs), suggest that persistent symptoms represent about $22.6 (95% CL: $13.0–$29.5) million, $0.20 per capita (95% CL: $0.12–$0.27), additional costs. These additional costs result in a total annual economic burden of dengue in Mexico of $192 (95% CL: $171–$325) million, a 13% increase over previous estimates ($170 million, $1.56 per capita, 2011–2012).^10^ Of these incremental costs, $2.0 million (95% CL: $0.6–$2.9) correspond to direct costs and $20.7 million (95% CL: $11.2–$27.5) correspond to indirect costs from productivity loss.

Considering only the acute burden, Undurraga and others^10^ estimated an annual disease burden of dengue episodes in Mexico (including age weights and time discounting) of 65.1 (95% CL: 36.0–98.7) disability-adjusted life years (DALYs) per million population. Using a disability weight of 0.219 (95% CL: 0.148–0.308, corresponding to “infectious disease, post-acute effects”), which is based on a combination from the Global Burden of Disease 2010 and the more recent European disability weight study^22^ and the predicted share of patients with persistent symptoms of dengue as a function of time, we found 28.2 (95% CL: 21.6–36.2) additional years lost to disability (YLD) per million population from persistent symptoms of dengue. Overall, including DALYs from acute dengue episodes as estimated by Undurraga and others^10^ and YLDs from persistent symptoms of dengue, we estimated that dengue imposes a total disease burden in Mexico of 93.3 (95% CL: 67.0–176.5) DALYs per million population annually, that is, a 43% increase over the previous estimate.^10^

Persistent symptoms were usually associated with female gender and older age.^7^,^11^,^13^,^15^,^16^ Although there is no clear understanding of how these characteristics lead to the persistence of symptoms, one possibility is that DENV generates a complex immunological response as a result of excess cytokine production during the acute phase,^23^ and it is possible that the interaction of the neuroendocrine, musculoskeletal, and immunological systems result in a persistent fatigue.^24^ Post-infection fatigue has also been found in Lyme disease, Epstein–Barr virus infection, and infectious mononucleosis,^7^ but the pathogenesis is not clearly understood.

This study has limitations to consider. First, the existing evidence does not allow robust conclusions about frequency, intensity, or duration of these sequelae of symptomatic DENV infection. Second, there is no clarity about the underlying physiopathological mechanisms of persistent symptoms following DENV infection, nor whether these symptoms are caused by dengue alone. Third, our estimates of additional economic and disease burden are based on parameters from a previous study, which also have limitations as acknowledged by the authors.^10^

Despite the uncertainty in our estimates, our results suggest that persistent symptoms of dengue illness may represent a substantial economic and disease burden that has not been elucidated previously. Valuing burden from this broader perspective resulted in about US$22.6 million in incremental costs and 28.2 incremental YLD annually. These represent a 13% increase on costs and a 43% increase in disease burden over previous estimates.^10^ Although broader than previous estimates of the burden of dengue, our numbers are still conservative. Other impacts of dengue are harder to measure, including the effects of outbreaks on tourism and travel, seasonal clustering of dengue on health systems, and several comorbidities and complications associated with dengue.^9^

Having accurate quantitative estimates of the disease burden of dengue is critical to set policy priorities and disease-control strategies, particularly as several vaccine candidates and other prevention and control technologies are currently under development. Mexico has a relatively high dengue incidence,^10^ and in December 2015 became the first country to license a dengue vaccine.^25^ Several authors have called for a more comprehensive evaluation of the burden of dengue.^8^,^9^ We hope that understanding the costs associated with persistent symptoms of dengue will improve previous estimates of the burden of dengue, and inform evidence-based health policy and priorities.

The study was approved by the institutional review boards of Brandeis University and the Instituto Nacional de Salud Pública, Cuernavaca, Mexico.

## Supplementary Material

Supplemental Datas.

## Figures and Tables

**Table 1:** Selected published literature related to persistent symptoms of illness following symptomatic DENV infection that may result in loss of productivity (asthenia, fatigue, trouble working)

Author	Country	Dengue definition	Follow-up	Sample size	Participants' age (years)	Prevalence of symptoms	RF and comments†
Teixeira and others^13^	Brazil	Clinically defined (86.5% laboratory confirmed)	2 weeks, 2 months, 6 months, 1 and 2 years	118	> 18	57%, 12%, and 5% reported difficulty to work at 2 weeks, 2 months, and 6 months, respectively	RF: female, age > 50 years. 65% reported symptoms at 2 weeks; 8.5% at 30 days.
Tristao and others^14^	Brazil	Laboratory confirmed	8, 15, 30, and 60 days	110 (90 DENV)	> 18	62%, 47%, 31%, and 20% at 8, 15, 30, and 60 days, respectively	No significant difference in persistence of symptoms by severity of illness
Dettogni and others^16^	Brazil	Laboratory confirmed	15, 30, 60 days	96	> 18	41%, 17%, and 5% at 15, 30, and 60 days, respectively	RF: female, secondary DENV infection
Del Valle and others^17^	Cuba	Laboratory-confirmed DHF	Monthly for 1 year	37	All ages, (62%: 15–34)	32%, 8%, and 5%, at 1, 2, and 3 months, respectively	Patients in sample had liver dysfunction from DENV
Garcia and others^11^	Cuba	Laboratory confirmed and asymptomatic (AD)	2 years	139	31–60	23% of symptomatic; 0% of AD	RF: female. No significant difference between severity and symptom persistence
Gonzalez and others^12^	Cuba	Laboratory-confirmed DHF/DSS	1, 2, 3, 4 weeks; 6 months	47	16–64	68%, 53%, 40%, 36%; and 27% at 1, 2, 3, 4 weeks and 6 months, respectively	Symptoms had irregular appearance, related to mental and physical activity
Lopez and others^18^	Cuba	Laboratory confirmed	1 year	28	18–48	36%, 11%, and 7% at 2 weeks, 6 months, and 12 months, respectively	Sample included only pregnant women
Halsey and others^15^	Peru	Laboratory confirmed	10–60 days	9,067 (3,659 DENV)	> 5	4%, 2%, and 2% at 10–20, 21–30, and 31–60 days, respectively	RF: older age and female gender. Passive follow-up, conservative estimates
Low and others^19^	Singapore	Laboratory confirmed	3 weeks	454 (133 DENV)	> 21	7%*	RT-PCR negative patients used as controls
Seet and others^7^	Singapore	Laboratory confirmed	2 months	127	16–70	24%	RF: older age, female, chills. Severity not significantly associated to fatigue

AD = asymptomatic dengue infection; DENV = dengue virus; DF = dengue fever; DHF = dengue hemorrhagic fever; DSS = dengue shock syndrome; RF = risk factors; RT-PCR = reverse transcriptase-polymerase chain reaction.

* 9.3% of patients presented with persistent symptoms after 3 weeks; of these, 72.7% reported fatigue.

† RFs relate to the patient showing any type of persistent symptom following a dengue episode. RFs are not necessarily associated to productivity loss.

**Table 2:** Summary of the incremental economic costs of persistent symptoms of dengue in Mexico (in millions of 2012 U.S. dollars)

	Direct costs*	Indirect costs†	Total
Persistent symptoms			
Subtotal	1.95	20.68	22.64
95% CL	0.63–2.88	11.22–27.53	13.01–29.45
Acute illness‡			
Hospitalized	22.56	2.71	25.27
Ambulatory	41.24	12.71	53.95
Fatal	–	7.57	7.57
Subtotal	63.80	22.99	86.79
95% CL	26.25–117.78	11.21–41.41	67.33–208.58
Surveillance and vector control§			82.92
Total annual costs∥	65.75	43.67	192.34
95% CL	41.59–165.93	35.12–95.44	170.64–325.25

CL = confidence level.

Based on estimates from a systematic literature review. 95% CL denotes 95% certainty level for the total estimates.

* Direct costs of persistent dengue symptoms assume one medication per month. The economic burden of dengue in Mexico by Undurraga and others^10^ was based on adjusted annual dengue episodes and vector control in 2010 and 2011.

† Indirect costs consider only adults, no children because of correlation between older age and persistent symptoms.

‡ The costs of acute symptoms of dengue are based on the estimates by Undurraga and others^10^.

§ Surveillance and vector control denotes the costs of surveillance and vector control based on the Ministry of Health annual budget.

∥ Total annual costs represent the estimated annual economic burden of dengue associated with persistent symptoms, acute illness, and surveillance and vector control costs. The 95% CL includes simultaneous variation of all parameters shown in Supplemental Table 2. On the basis of the regression results (Figure 1), we assumed that no patients had work-limiting symptoms after 11 months.
