# Supplementary material for: Persistent Symptoms of Dengue: Estimates of the Incremental Disease and Economic Burden in Mexico
Source: Am J Trop Med Hyg. 2016 May 4;94(5):1085–9. doi: 10.4269/ajtmh.15-0896 (PMC4856607; doi:10.4269/ajtmh.15-0896)
Supplement: Supplementary file 1 [file SD5.pdf]

SUPPLEMENTAL MATERIAL

SYSTEMATIC LITERATURE REVIEW

We looked for persistent symptoms of dengue fever from a variety of sources. We performed a systematic literature review of articles published or indexed on PubMed, MEDLINE, SciELO, and the World Health Organization (WHO) Dengue Bulletin combining the keyword "dengue" with each of the following: fatigue, chronic, persist*, post-infect*, long-term, and clinical symptom*, for years 1995 through October 2015, in English, Spanish, French, and Portuguese (* indicates that additional letters are optional). We selected all articles related to persistent symptoms of dengue that had full text available, empirical data on persistent symptoms of dengue which may result in loss of productivity, a scientifically valid approach, and external validity. We excluded reviews, editorials, purely subjective papers, opinions, and duplicated studies.

We identified a total of 68 (of 2,221) articles related to long-term consequences of dengue fever for review, plus one additional article from reviewing the references. Of these, 10 articles (from Brazil, Cuba, Peru, and Singapore) satisfied our inclusion criteria of having a full text available, empirical data on potential work loss, scientifically valid approach, and external validity (Supplemental Table 1 and Supplemental Figure 1).

Probabilistic sensitivity analysis. To address uncertainty in our economic and disease burden estimates, we used a probabilistic sensitivity analysis. We allowed for variation in the main parameters used by Undurraga and others[2] (i.e., expansion factors, direct medical costs, direct nonmedical costs, health service utilization, patient impact, and household impact), and allowed for uncertainty in the loss of productivity and additional expenses in medications and diagnostic tests, which might have resulted from persistent symptoms, as described in the main manuscript. We computed 10,000 Monte Carlo simulations based on the simultaneous variation of all parameters. Supplemental Table 2 shows the specific parameters, distributions, and values for the sensitivity analysis.

SUPPLEMENTAL REFERENCES

1. Moher D, Liberati A, Tetzlaff J, Altman DG, 2009. Preferred reporting items for systematic reviews and meta-analyses: the PRISMA statement. *PLoS Med 6:* e1000097.
2. Undurraga EA, Betancourt-Cravioto M, Ramos-Castañeda J, Martínez-Vega R, Méndez-Galván J, Gubler DJ, Guzmán MG, Halstead SB, Harris E, Kuri-Morales P, Tapia-Conyer R, Shepard DS, 2015. Economic and disease burden of dengue in Mexico. *PLoS Negl Trop Dis 9:* e3547.
3. Reynolds KJ, Vernon SD, Bouchery E, Reeves WC, 2004. The economic impact of chronic fatigue syndrome. *Cost Eff Resour Alloc 2:* 4.
4. Lin J-MS, Resch SC, Brimmer DJ, Johnson A, Kennedy S, Burstein N, Simon CJ, 2011. The economic impact of chronic fatigue syndrome in Georgia: direct and indirect costs. *Cost Eff Resour Alloc 9:* 1.
5. Salomon JA, Haagsma JA, Davis A, de Noordhout CM, Polinder S, Havelaar AH, Cassini A, Devleesschauwer B, Kretzschmar M, Speybroeck N, 2015. Disability weights for the Global Burden of Disease 2013 study. *Lancet Glob Health 3:* e712–e723.
6. Shepard DS, Coudeville L, Halasa YA, Zambrano B, Dayan GH, 2011. Economic impact of dengue illness in the Americas. *Am J Trop Med Hyg 84:* 200–207.
7. World Health Organization, 2011. Choosing interventions that are cost effective (WHO - CHOICE). Available at: http://www.who.int/choice/en/. Accessed January 4, 2011.
8. Secretaría de Salud México, 2013. Sistema nacional de información en salud. Available at: http://www.sinais.salud.gob.mx. Accessed November 1, 2012.
9. Secretaría de Desarrollo Social, 1999. Sistema normativo de equipamiento urbano. Tomo II: salud y asistencia social. Mexico, DF: Dirección General de Infraestructura y Equipamiento.
10. Suaya JA, Shepard DS, Siqueira JB, Martelli CT, Lum LCS, Tan LH, Kongsin S, Jiamton S, Garrido F, Montoya R, Armien B, Huy R, Castillo L, Caram M, Sah BK, Sughayyar R, Tyo KR, Halstead SB, 2009. Cost of dengue cases in eight countries in the Americas and Asia: a prospective study. *Am J Trop Med Hyg 80:* 846–855.

Supplemental Table 1

Total records identified through PubMed, MEDLINE, SciELO, and WHO Dengue Bulletin, and articles assessed for eligibility by search criteria

Search criteria "Dengue" plus	PubMed	MEDLINE†	SciELO	Dengue bulletin
Fatigue	4 (30)	5 (29)	1 (4)	1 (5)
Chronic	4 (145)	4 (114)	0 (11)	2 (18)
Persist*	11 (282)	13 (269)	5 (36)	2 (58)
Post-infect*	4 (102)	3 (99)	0 (14)	0 (0)
Long-term	5 (166)	4 (159)	1 (6)	3 (158)
Clinical symptom*	5 (86)	6 (84)	13 (85)	11 (261)

The numbers in parenthesis show the total number of entries for each combination of keywords in each database. Of the 107 selected articles, 39 were repeated.

*Indicates that additional letters are optional.

†Medline search was limited to words appearing in the abstract.

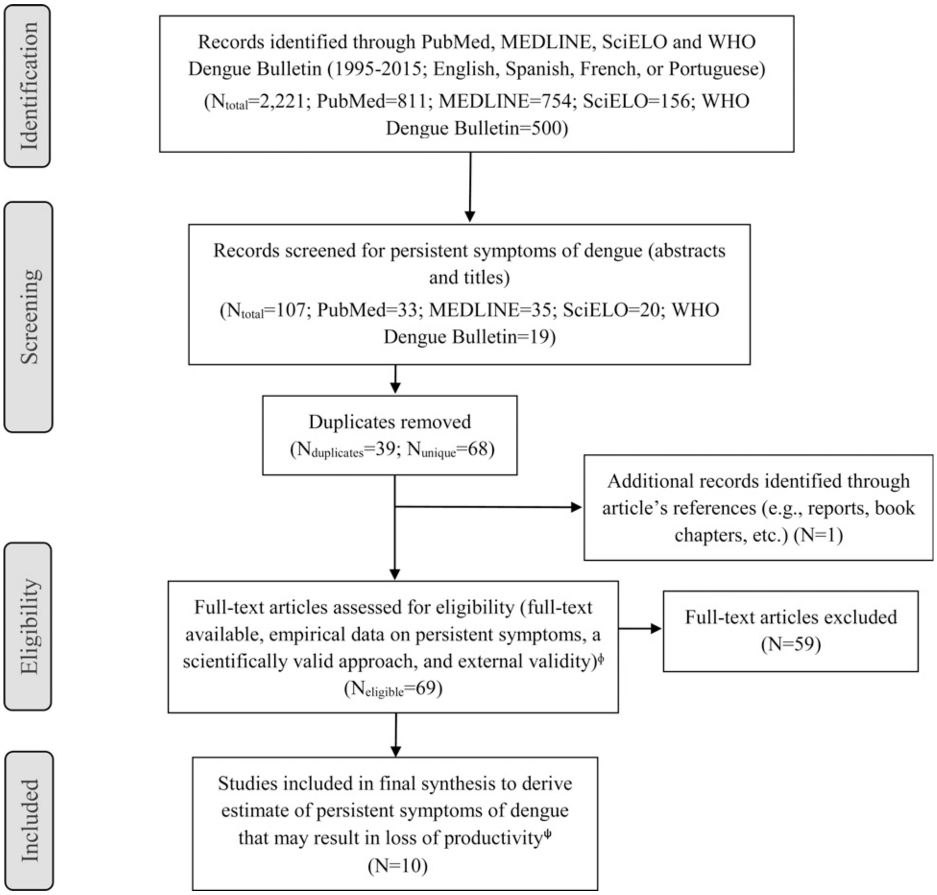

SUPPLEMENTAL FIGURE 1. Systematic literature review summary diagram. PRISMA flow diagram based on Moher and others.[1] ᴪPersistent symptoms that may cause loss of productivity include fatigue, asthenia, and reporting difficulty to work. ᶲThe number of articles that did not comply with our eligibility criteria were two did not have full text available, 44 did not report empirical data on persistent symptoms of dengue, which may result in loss of productivity, three did not use a scientifically valid approach that provided reliable data (e.g., provided anecdotal evidence), and 17 lacked sufficient external validity (mostly due to dengue comorbidities). Some articles lacked more than one eligibility criteria.

Supplemental Table 2

Summary of the parameters varied simultaneously for 10,000 Monte Carlo simulations in the probabilistic sensitivity analysis

Item	Units	Estimate	Distribution	Statistics	Value	Source
Persistent symptoms						
Monthly expenditures in medications and diagnostics	$	10.52	Beta-PERT	(Min; best; max)	(0.00; 10.52; 17.23)	Undurraga and others[2]
Loss of productivity	%	45	Beta-PERT	(Min; best; max)	(15; 45; 65)	Reynolds and others,[3] Lin and others[4]
Disability weights		0.219	Beta-PERT	(Min; best; max)	(0.148; 0.219; 0.308)	Salomon and others[5]
Economic and disease burden of dengue in Mexico (Undurraga and others[2])						
Expansion Factors						
Hospitalized	EF_H	2.0	Beta-PERT	(Min; best; max)	(1.0; 2.0; 3.3)	Morelos cohort, Shepard and others[6]
Ambulatory	EF_A	5.6	Beta-PERT	(Min; best; max)	(5.0; 5.6; 15.0)	Morelos cohort, Shepard and others[6]
Direct medical costs						
Hospitalized	$	238.91	Beta-PERT	(Min; best; max)	(229.5; 238.9; 309.5)	Macro-costing, WHO,[7] MoH[8]
Ambulatory	$	65.25	Beta-PERT	(Min; best; max)	(17.23; 65.3; 99.1)	Macro-costing, WHO,[7] MoH,[8,9] Morelos cohort, interviews
Direct nonmedical costs						
Hospitalized adults	$	25.16	Normal	(μ, σ)	(25.2; 7.0)	Patient interviews
Ambulatory adults	$	11.96	Normal	(μ, σ)	(12.0; 8.3)	Patient interviews
Hospitalized children	$	27.85	Normal	(μ, σ)	(27.8; 6.4)	Patient interviews
Ambulatory children	$	9.09	Normal	(μ, σ)	(9.1; 2.0)	Patient interviews
Duration of episode (acute + convalescent phase)						
Hospitalized	Days	13.9	Normal	(μ, σ)	(13.9; 5.3)	MoH surveillance, Suaya and others[10]
Ambulatory	Days	12.3	Normal	(μ, σ)	(12.3; 5.4)	MoH surveillance, Suaya and others[10]
Health service utilization						
Hospitalized	Days	3.5	Normal	(μ, σ)	(3.5; 4.3)	MoH surveillance
Ambulatory (prehospital, adult)	Days	2.4	Normal	(μ, σ)	(2.4; 1.1)	Patient interviews
Ambulatory (prehospital, child)	Days	3.7	Normal	(μ, σ)	(3.7; 2.5)	Patient interviews
Ambulatory	Days	3.9	Normal	(μ, σ)	(3.9; 2.1)	Suaya and others[10]*
Patient impact (average days lost by patient)						
Hospitalized, school loss	Days	6.2	Normal	(μ, σ)	(6.2; 4.2)	Suaya and others[10]*
Ambulatory, school loss	Days	4.4	Normal	(μ, σ)	(4.4; 3.3)	Suaya and others[10]*
Hospitalized, work loss	Days	9.8	Normal	(μ, σ)	(9.8; 4.3)	Suaya and others[10]*
Ambulatory, work loss	Days	5.4	Normal	(μ, σ)	(5.4; 4.3)	Suaya and others[10]*
Household impact (average days lost by each household member affected)						
Hospitalized, school loss	Days	3.7	Normal	(μ, σ)	(3.7; 4.5)	Suaya and others[10]*
Ambulatory, school loss	Days	2.2	Normal	(μ, σ)	(2.2; 3.5)	Suaya and others[10]*
Hospitalized, work loss	Days	6.1	Normal	(μ, σ)	(6.1; 6.6)	Suaya and others[10]*
Ambulatory, work loss	Days	3.8	Normal	(μ, σ)	(3.8; 5.3)	Suaya and others[10]*

EF = expansion factor; MoH = Ministry of Health; PERT = Project Evaluation and Review Techniques; WHO = World Health Organization. The main parameters used to estimate the burden of dengue in Mexico and their rationale are extensively described in Undurraga and others.[2] The normal distributions for medical expenditures and days lost were lower-truncated at zero. Beta-PERT distributions (lower bound, mode, upper bound, and λ) with $\lambda = 4$.

*Simple average from countries in the Americas included in the work of Suaya and others,[10] that is, Brazil, El Salvador, Guatemala, Panama, and Venezuela.
